# Enhanced view/extended totally extraperitoneal plasty (eTEP) Rives–Stoppa repair versus open Rives–Stoppa repair: a single-center retrospective propensity score-matched cohort study

**DOI:** 10.1007/s00464-026-13049-0

**Published:** 2026-07-02

**Authors:** Robin Klewitz, Nico Pfander, York Gruenewald, Hans Christian Hillebrecht, Stefan Fichtner-Feigl, Philipp Anton Holzner, Julian Hipp

**Affiliations:** Department of General and Visceral Surgery, Medical Center, University of Freiburg, Hugstetter Str. 55, 79106 Freiburg, Germany

**Keywords:** eTEP, Rives–Stoppa repair, Retromuscular repair, Ventral hernia repair, Minimally invasive surgery

## Abstract

**Background:**

To evaluate the comparative effectiveness of extended totally extraperitoneal plasty Rives–Stoppa retromuscular repair (eTEP-RS) and open Rives–Stoppa retromuscular repair (Open-RS) in patients undergoing ventral hernia repair.

**Methods:**

Clinical data from 850 patients were collected in a prospectively maintained database and retrospectively evaluated. 153 patients undergoing eTEP-RS were compared to 154 selected patients undergoing Open-RS (from the period prior to implementation of eTEP-RS at our university medical center). Short-term perioperative outcomes as well as long-term recurrence rate and quality of life by Carolina Comfort Scale (QoL) were evaluated. Results are shown as median (interquartile range).

**Results:**

Our learning curve phase (first 60 eTEP-RS cases) was compared to eTEP-RS from the steady-state phase (cases 61–153). Significant differences with regard to operation time and perioperative complications were observed indicating a relevant learning curve in the procedure. The eTEP-RS cases from the steady-state cohort were compared to the Open-RS cases. After propensity score matching, 83 eTEP-RS cases were compared to 83 Open-RS cases. While operation time was longer (Open-RS: 135 min (95–161); eTEP-RS: 160 min (126–192); *i* = 0.004), length of stay was shorter in the eTEP-RS cohort (Open-RS: 7 days (6–9); eTEP-RS: 4 days (3–5); *p* < 0.001) and postoperative pain was lower on postoperative days 2 and 3. Perioperative complications, hernia recurrence rates, and long-term QoL were not different between the two cohorts.

**Conclusion:**

eTEP Rives–Stoppa repair offered superior short-term outcomes compared to open Rives–Stoppa repair in suitable patients with medium-sized ventral hernia and selected patients with large ventral hernia. Given the short follow-up period, no statistically significant differences could be observed regarding the long-term outcomes recurrence rate and QoL. Future long-term multicenter studies are necessary to evaluate long-term efficacy.

**Graphical Abstract:**

**Figure:**
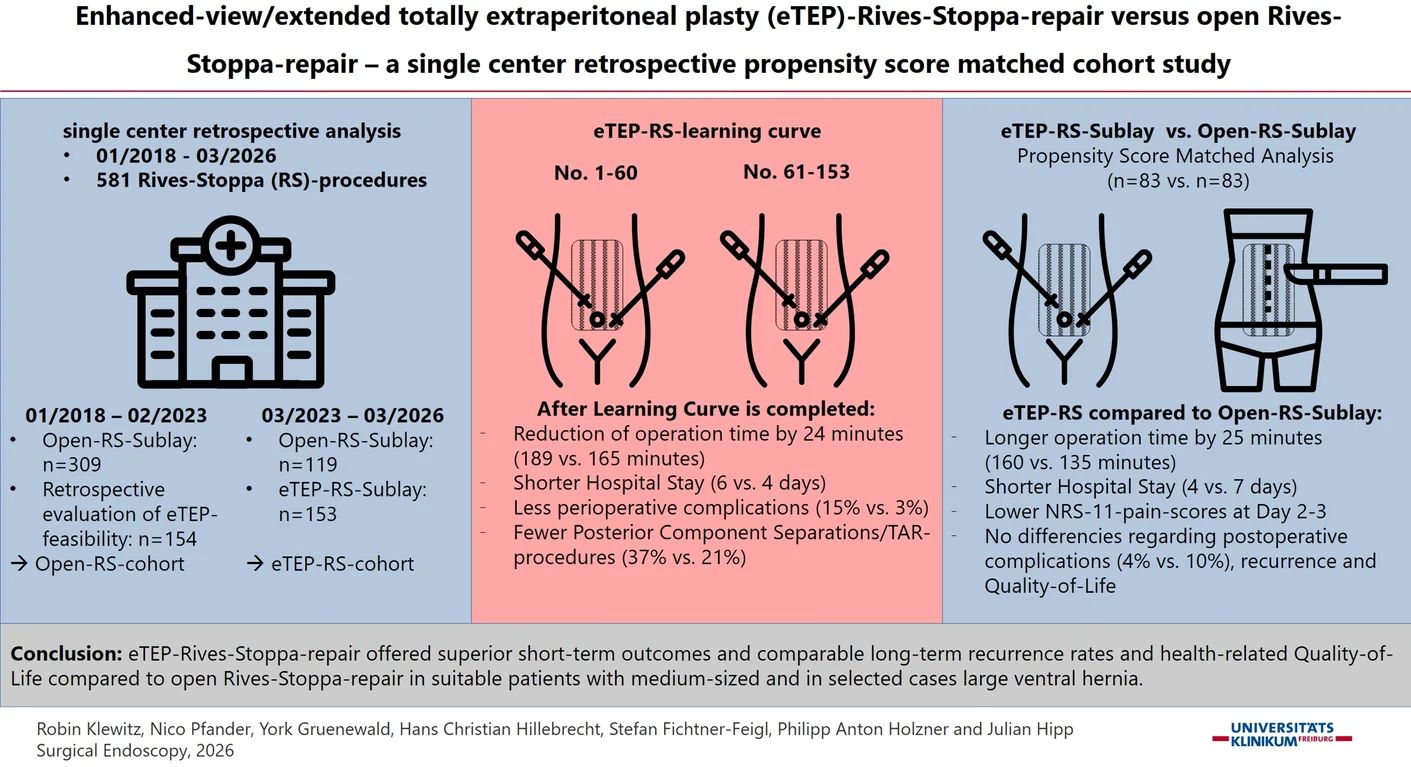

**Supplementary Information:**

The online version contains supplementary material available at https://doi.org/10.1007/s00464-026-13049-0.

Ventral hernias and incisional hernias are a relevant socio-economic health care-associated problem. Despite significant progress in the surgical techniques for the closure of abdominal incisions, incisional hernias still occur after 10–20% of all laparotomies [1]. This leads to ~50,000 surgical repairs of incisional hernia performed annually with an incidence of 104,000/year in Germany alone [2]. In the past, hernia surgery was associated with substantial long-term morbidity by a high recurrence rate [3]. With the ground-breaking development of the mesh-augmented retromuscular repair by Rives and Stoppa, a surgical technique with favorable short- and long-term results was established as a gold standard for ventral hernia repair [4–6]. In the 1990s, laparoscopic access to ventral hernia surgery was introduced and the intraperitoneal onlay mesh (IPOM) technique gained widespread acceptance and was (and still is) the main access for ventral hernia repair in many hernia centers due to its favorable short-term results [7]. However, the IPOM procedure is associated with postoperative pain due to mesh fixation and an inherent risk for long-term mesh-associated complications due to the intraperitoneal position of the mesh [8]. This led to a paradigm shift in recent years with the revival of the usage of the retromuscular plane as the optimal space for mesh placement in ventral hernia repair [9].

This development was supported by the establishment of minimally invasive techniques which reproduce the anatomy of the open Rives–Stoppa retromuscular repair (Open-RS) in a minimally invasive fashion [10, 11]. At the forefront of this development, the enhanced view/extended totally extraperitoneal plasty (eTEP) Rives–Stoppa retromuscular repair (eTEP-RS) is the most promising technique for laparoscopic and robotic-assisted ventral hernia repair due to its fully minimally invasive technique [12–16]. The eTEP-RS procedure has been proven to be a feasible and safe technique for medium to large ventral hernias [17].

Retrospective analysis could show that a combination of a mini- or less-invasive approach with extraperitoneal mesh placement offers superior long-term results regarding risk of recurrence, chronic pain, and general complications compared to laparoscopic IPOM repair [18]**.** While several studies have investigated the comparative effectiveness and outcomes between IPOM and eTEP-RS repair [19], studies comparing eTEP-RS with its open surgical predecessor, the Open-RS procedure, are rare with only small cohorts of patients [20–22].

Due to the given long-term risks of intraperitoneal mesh placement, the standard treatment for ventral hernia repair was always a retromuscular technique at our university medical center. IPOM procedures were only used in rare cases when retromuscular repair was not feasible due to impaired abdominal wall anatomy. After the first eTEP-RS procedure was performed in 02/2023 at our department, we quickly saw the advantages for the patients and made it our standard approach for ventral and especially incisional hernia repair. The main indication for eTEP-RS at our department is ventral hernia/incisional hernia 3–8 (− 10) cm in width (W2 according to the EHS classification [23]). Larger defects > 10 cm were routinely managed using the Open-RS approach (and additional component separation techniques if necessary) and eTEP-RS was only used in selected patients. No specific contraindications were applied regarding transverse incisions (including inverted L-incision for liver surgery (Makuuchi incision)) and length of the incision (including expected difficult crossover maneuver due to M1-5 incision). eTEP-RS became the standard treatment for medium-sized hernia at our department, as long as the hernia defect seemed to be manageable by the approach. Our cohort of patients is therefore ideally suited for the comparison of Open-RS and eTEP-RS. The aim of this study was therefore to compare eTEP-RS and Open-RS procedure regarding short- and medium- /long-term outcomes at our tertiary university medical center.

## Methods

**Fig. 1:**
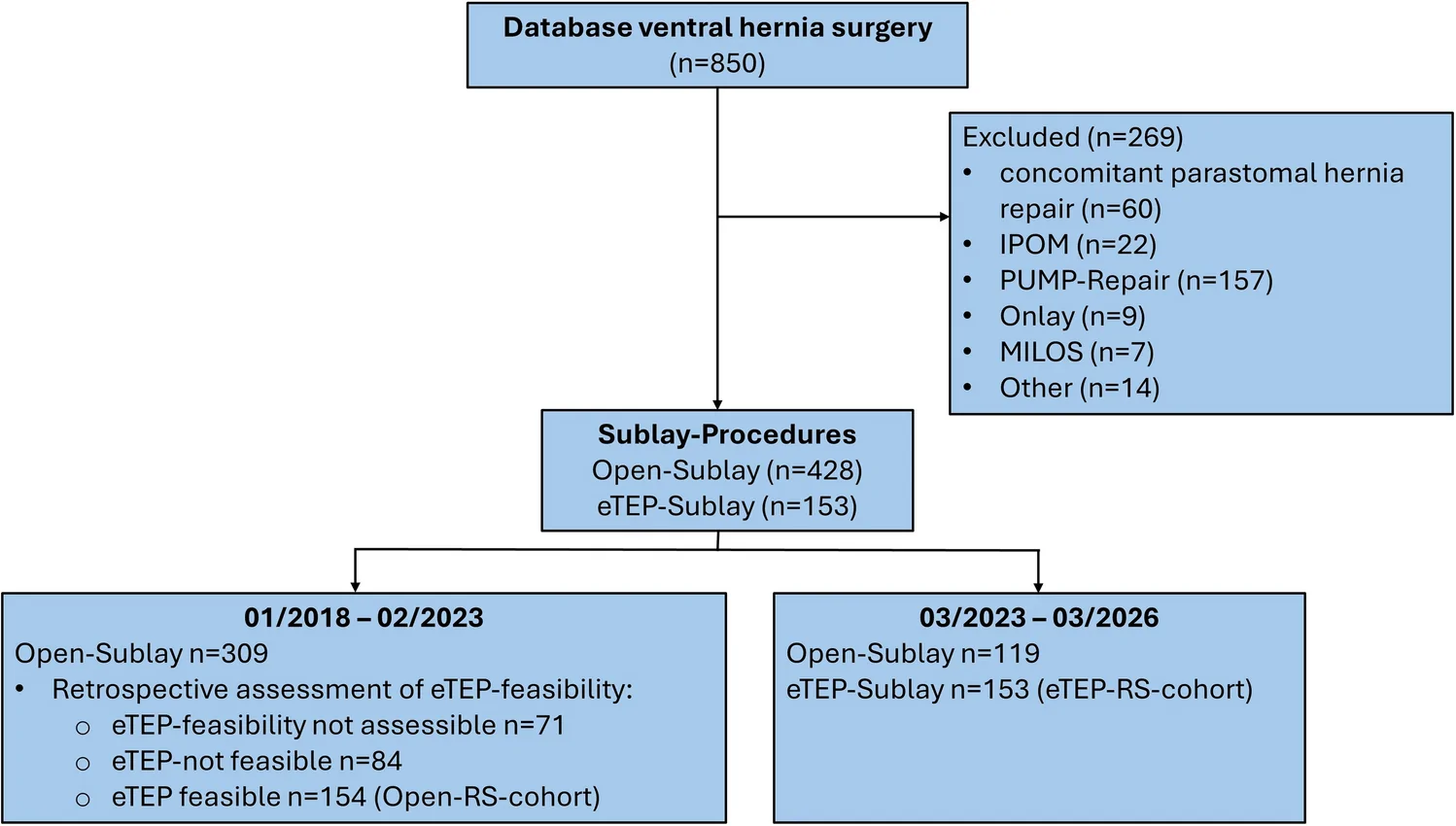
Study flow chart

This study is reported in accordance with the 2025 STROCSS statement (STROCSS statement available: Supplementary Data) [24, 25]. Patient data from 01/2018 to 03/2026 were collected in our database for hernia surgery at our university medical center (DRKS00037048; German Clinical Trials Register: https://www.drks.de) and evaluated retrospectively. According to the a priori study protocol (available as supplementary data, patient representatives were not involved in the study design), all consecutive patients undergoing ventral hernia repair in the mentioned study period were included in this single-center cohort study. Of 850 patients with ventral hernia, 581 patients were treated with retromuscular repair by either Open-RS or eTEP-RS and therefore met the inclusion criteria for this study. 269 patients were excluded due to concomitant parastomal hernia repair (*n* = 60), IPOM repair (*n* = 22), PUMP repair (*n* = 157), Onlay repair (*n* = 9), MILOS repair (*n* = 7), and other procedures (*n* = 14). (Fig. 1). The study was reviewed and approved by the Ethics Committee of the University of Freiburg, Germany (25-1341-S1 and 25-1498-S1-retro).

### Procedures

Open Rives–Stoppa retromuscular repair (Open-RS) was performed as previously described [26, 27]. The patient is positioned in supine position, general anesthesia is induced. The old scar is excised and the hernia sac is dissected from subcutaneous tissue. The hernia defect and the fascial margins are dissected and the retromuscular plane is entered bilaterally. The preparation is circumferentially performed until a mesh overlap of at least 5 cm in all directions is achieved. This includes retroxyphoidal preparation and retropubic preparation in the space of Retzius as needed. Posterior component separation (transversus abdominis release [28, 29]) is performed as necessary for fascial closure or management of lateral defects or defects crossing the EIT ambivium [30]. After that, the posterior layer is reconstructed using 2–0 PDS suture and a properly sized synthetic mesh is placed in the retromuscular space. The mesh is fixated in all cases with several transfascial 2–0 PDS sutures using an EndoClose Needle (Medtronic, Dublin, Ireland). Retromuscular drains are placed routinely in Open-RS cases. Finally, the linea alba is reconstructed using a size 2–0 or 1 PDS suture and the subcutaneous tissue and the skin are closed. Excess skin is resected, if necessary. All Open-RS procedures were either directly performed or supervised by a general surgeon specialized in abdominal wall reconstruction.

**Fig. 2:**
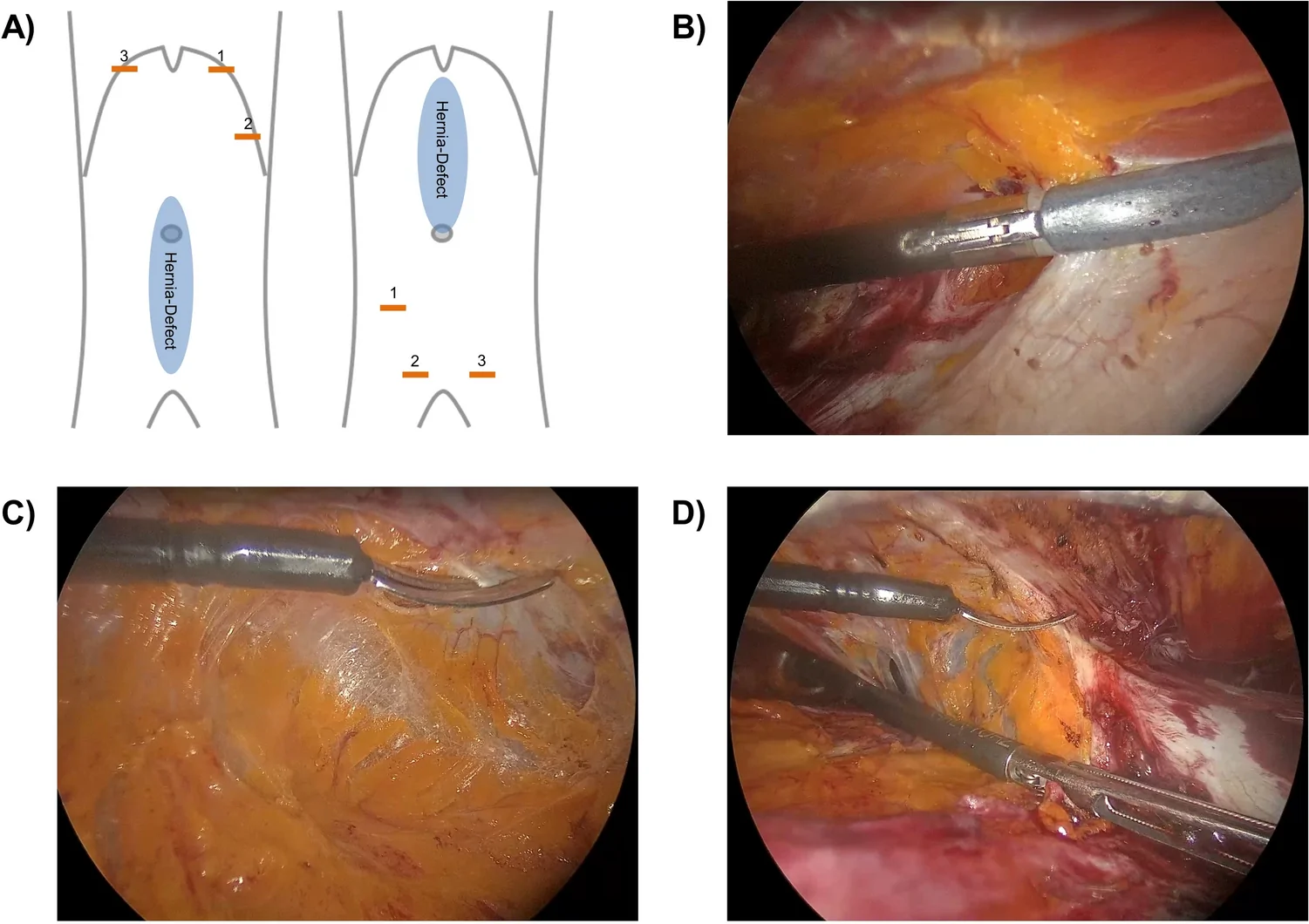
Trocar placement for laparoscopic eTEP Rives–Stoppa repair and superior cross over maneuver. **A** Laparoscopic eTEP Rives–Stoppa repair has two placement options for the trocars: if the hernia defect is located caudally, three trocars are placed along the costal margin and the left EIT Ambivium. For cranial hernia defects, the trocars are placed along the right EIT Ambivium and suprapubically in the left retrorectus space. The initial camera trocar (1) is 12 mm, and the initial working trocar (2) is 5 mm. After the crossover maneuver is performed, the camera is placed in trocar 3 (12 mm) for the rest of the procedure, while trocars 1 and 2 are used as working ports. For large defects, a 5–6 trocar setting combining both setups can be used. **B** The superior crossover maneuver is initiated by incising the posterior rectus sheath of the left retrorectus space ~5 mm dorsally to the linea alba. The dissection is continued between the linea alba and the falciform ligament. **C** The right posterior rectus sheath is visualized and incised to connect both retrorectus spaces. **D** After that, the camera is placed in the right retrorectus space and the dissection is continued in a caudal direction toward the hernia defect

eTEP Rives–Stoppa retromuscular repair (eTEP-RS) was performed according to previous descriptions of the technique either in laparoscopic or robotic-assisted technique [13, 15, 31–33] (Fig. 2). In summary, the patient is positioned in supine position, general anesthesia is induced and a Foley catheter is placed routinely for the procedure. After that, the operating table is flexed around 20°. Two surgical setups are available for the laparoscopic approach: For caudal (M2/3-5) hernia, the superior crossover setup is used with three trocars along the costal margin and the left EIT-ambivium, and for cranial hernias (M1-2), the bottom crossover setup is used with three trocars in the suprapubic region and along the right EIT ambivium. A combined setup with 5–6 trocars is possible for bilateral transversus abdominis release or hernias that need extended longitudinal dissection (e.g., retroxyphoidal and retropubic). After that, eTEP-RS follows the principles of the following:

1. Development of one-sided retrorectus space and port placement.
2. Cross-over of the midline (ideally) above/below the hernia defect anterior to the falciform ligament/in the space of Retzius.
3. Connection of both retrorectus spaces.
4. Defect closure and restoration of the linea alba and the posterior layer.
5. Mesh placement.

In the subxyphoidal region, the plane of preparation is changed from the retromuscular plane to the preperitoneal plane by transverse incision of the posterior layer of the rectus sheath, according to the Madrid modification of the TAR access. With this modification, the medialized cranial neurovascular bundles can be spared and functional results for the patients may be optimized [34, 35]. If necessary, retroxyphoidal preparation is carried out in a preperitoneal subdiaphragmal plane toward the central tendon of the diaphragm.

The necessity for posterior sheath reconstruction and its impact on the postoperative result remain a discussed topic among eTEP-surgeons [36, 37]. We use a pragmatic approach in this regard. If posterior sheath reconstruction is possible without undue tension, we reconstruct the posterior layer of the rectus sheath due to its possible positive effects on postoperative results. In many cases with small rectus-to-defect ratio (RDR) or larger incisional hernia, reconstruction of the posterior layer will not be possible without the risk of postoperative posterior sheath defect. In these cases, we do not reconstruct the posterior sheath and bridge the posterior layer defect by preperitoneal tissue or a hernia sac-bridging technique [38]. Especially the use of the hernia sac-bridging technique can effectively reduce the necessity for TAR for posterior layer reconstruction, which is a known problem of the eTEP-RS-technique [39].

Posterior component separation using the transversus abdominis release (TAR) technique is used as necessary [40, 41]. In routine-eTEP-RS, mesh width of 10–15 cm (within the confinements of the retrorectus space) is strived for after complete anterior fascial closure [42]. In TAR cases, wider and larger meshes are used within the entire dissected retromuscular space. In the rare case of mesh fixation, transfascial sutures were used. All eTEP procedures were either directly performed or supervised by one of two specialized abdominal wall surgeons (J.H. and R.K.).

### Perioperative management

In Open-RS cases with larger medium-sized and large ventral hernia, peridural catheters were placed, if no contraindications were present. In eTEP-RS cases, peridural catheter was not deemed necessary, as we observed less pain in the first treated patients. We therefore only used more invasive pain treatment with peridural anesthesia in patients with large (W3) hernia and in case of patients requesting peridural anesthesia. Despite that, all patients were treated according to the WHO scheme for pain management, mostly with a non-opioid-analgesic and a low-to-medium-potency oral opioid (Tilidine) with i.v. bolus administration of piritramide as necessary [43]. Retromuscular and subcutaneous drains were routinely placed in Open-RS cases. In eTEP-RS cases, retromuscular drains were only placed in patients with risk factors for hematoma in the beginning, but after a few necessary surgical revisions for hematoma in the learning curve phase, we decided to place drains in most patients again.

All patients were advised to wear an abdominal binder in the first 4 weeks after surgery. Discharge was possible after removal of all drains (drain output < 50–100 cc/day), sufficient pain control with oral analgesic medication, and sufficient mobilization of the patient.

### Cohorts

We treated all feasible patients with eTEP-RS from 03/2023 on, so this eTEP-RS cohort cannot be compared to the Open-RS patients from the same period, as these patients did not undergo eTEP-RS due to anatomic/hernia-related reasons.

Our current criteria for using the eTEP-RS approach instead of an Open-RS approach are as follows:

#### Inclusion criteria for eTEP-RS

- Median hernia (primary ventral hernia and incisional hernia) 3–8 cm width
- Median hernia ≥ 8 cm width: component separation index (CSI) ≤ ~0.2 (70°/360°), whereby we anticipate necessity of TAR in a CSI range between 0.12–0.15 and 0.2 [44]. The RDR is also evaluated in these cases, necessity of TAR is highly likely in cases with RDR < 1.5 to 2 [45]
- Ventral hernia < 3 cm width with relevant/symptomatic diastasis recti
- Incisional hernia after transverse incision/Makuuchi incision with cranio-caudal defect length ≤ 6 cm, transversal defect width does not matter in these cases.

#### Exclusion criteria for eTEP-RS

- Ventral hernia < 3 cm width without relevant concomitant diastasis recti
- Narrow rectus sheath ≤ 4 cm (one sided)
- Loss of domain ≥ 20% Sabbagh’s method [46]
- Concomitant enterocutaneous fistula/soft tissue/mesh infection
- Recurrent hernia after previous Rives–Stoppa sublay repair
- Need for extensive scar excision (hybrid-eTEP with local scar excision in selected cases possible)

Of course, these criteria have to be seen as guidelines and individualized decisions on eTEP feasibility have to be made in every single patient in routine clinical practice. Complex hernias according to the EHS consensus were only included in cases of width ≥ 10 cm, BMI ≥ 40 kg/m^2^, and liver cirrhosis with ascites. Other reasons for complexity (e.g., loss of domain; presence of skin defects/ulceration; presence of fistula; current mesh infection) were not considered for eTEP-RS procedures [47].

Since comparison with the current Open-RS cohort was not possible as mentioned above, we therefore reviewed all 309 patients (Review by J.H. and R.K.) undergoing Open-RS in the period from 01/2018 to 02/2023 retrospectively by analyzing preoperative CT scans/MRI scans and clinical data for retrospective feasibility of eTEP-RS in this cohort. In 71 patients, the feasibility of eTEP could not be assessed retrospectively; in 84 patients, eTEP-RS was not deemed feasible; and in 154 patients, we considered eTEP-RS feasible (Supplementary Data Table S1). These 154 patients are the Open-RS control cohort, to which the eTEP-RS cohort was compared in this study (Fig. 1). The eTEP-RS cohort was split into an eTEP-RS learning cohort (case 1–60) and an eTEP-RS steady-state cohort (case 61–153). To avoid learning curve contamination of the primary analysis of this study, the comparison of eTEP-RS and Open-RS with respect to short-term outcomes, only the eTEP-RS steady-state cohort was compared to the Open-RS cohort for the final subgroup analysis.

### Data extraction

The following data were extracted from our database: demographic data: age, gender, body mass index (BMI), RCS Charlson comorbidity index [48], American Society of Anesthesiologists (ASA) physical status (ASA score) [49], smoking history, and anticoagulation medication. Hernia specific data: type of hernia (primary ventral hernia, incisional hernia, parastomal hernia), EHS classification (all defects (incisional and primary hernia) were classified according to the EHS classification for incisional hernia for data homogeneity) [23], size of the hernia defect (width x length), and defect area in cm^2^ according to EHS classification [23]. Procedural data: type of procedure, operation time, mesh type, mesh size, mesh-to-defect ratio (MDR), and mesh fixation. Postoperative data: numerical rating scale for pain (NRS-11 score) [50] as patient-reported outcome on postoperative days (POD) 1–3 and on discharge (maximum value of each day at rest and during exercise), peridural catheter placement, pain medication (opioids) at discharge, time to first bowel movement, drainage volume and days with drainage, length of intensive care/intermediate care unit (ICU) stay (days), necessity for ICU readmission, length of hospital stay, 30-day readmission rate, 30-day Clavien–Dindo classification [51], 30-day surgical site occurrence requiring procedural intervention (SSOPI) rate, long-term follow-up including hernia recurrence according to Lima et al. [52], and quality of life assessed by the Carolina Comfort Scale [53].

### Statistical analysis

Follow-up data including survival and hernia recurrence were systematically obtained until April 2026. The median follow-up was 17 months (48 months Open-RS; 7 months eTEP-RS) calculated with reverse Kaplan–Meier method [54]. Post hoc power calculation regarding non-inferiority for hernia recurrence showed a statistical power of 0.36 at a one-sided *p* = 0.05 and a non-inferiority margin of 0.1, a 2-year follow-up, and 5% vs. 7% recurrence rates [55]. Recurrence rates were calculated using an Aalen–Johansen estimator and Fine–Grey analysis for competing risk analysis [56, 57].

To adjust for differences in the control cohort and eTEP steady-state cohort, propensity score matching with 1:1 nearest neighbor method with a matching caliper of 0.2-standard deviations was performed [58] with prespecified parameters (age, body mass index (BMI), RCS Charlson comorbidity index [48], hernia defect area in cm^2^, location (M1-5, L1-4), and hernia width (W status) according to EHS classification [23]). Post hoc power analyses to demonstrate superiority of eTEP-RS vs. Open-RS with regard to severe complications (≥ Grade IIIb) or SSOPI was 0.33 at a two-sided *p* = 0.05 in the propensity score-matched cohort [55].

All analyses were performed as an intention-to-treat analysis. Results are expressed as median (interquartile range) or as number (percent). We used Mann–Whitney *U*-test for descriptive analysis of non-parametric variables and Pearson’s chi-squared test/Fisher’s exact test for categorical variables. Statistical analysis was performed using SPSS version 31.0.0.0 (IBM Corp., Armonk, NY, USA) and R version 4.5.2 (R Foundation for Statistical Computing, Vienna, Austria) with R Studio 2026.01.0 (R Studio Inc., Boston, MA, USA) and additional packages tidycmprsk, ggsurvfit, and ggplot2. Differences were considered statistically significant for *p* < 0.05.

## Results

### Study cohorts

Details regarding the patient cohorts are outlined in Tables 1, 2, 3 and 4 and Supplementary Data Tables S2 and S3. 307 patients were included in the final study cohorts (Fig. 1). The control cohort (Open-RS cohort) consisted of 154 patients undergoing Open-RS, and the intervention cohort consisted of 153 patients undergoing eTEP-RS. Patient data comparing all these patients are shown in Supplementary Data Tables S2 and S3.

**Table 1:** Learning curve of eTEP cases, demographic data

	First 60 eTEP-RS cases (*n* = 60)	eTEP-RS cases 61–131 (*n* = 93)	*p*-value
*Age*	65 (58–70)	63 (50–72)	0.734*
Sex			
Male	36 (60%)	55 (59%)	0.916^#^
Female	24 (40%)	38 (41%)	
*BMI*	27.2 (23.5–33.8)	29.0 (24.7–32.4)	0.370*
RCS Charlson score			
0	17 (28%)	26 (28%)	0.490^#^
1	22 (37%)	34 (37%)	
2	18 (30%)	22 (24%)	
≥ 3	3 (5%)	11 (12%)	
ASA classification			
ASA 1	1 (2%)	1 (1%)	0.288^#^
ASA 2	15 (25%)	26 (28%)	
ASA 3	40 (67%)	65 (70%)	
ASA 4	4 (7%)	1 (1%)	
Anticoagulation			
Yes	7 (12%)	13 (14%)	0.808^#^
Type of hernia			
Primary ventral hernia	11 (18%)	20 (21%)	0.685^#^
Incisional hernia	49 (82%)	73 (79%)	
*Recurrent hernia*	6 (10%)	10 (11%)	1.000^#^
EHS classification			
M1	11 (18%)	22(24%)	0.547^#^
M2	39 (65%)	66 (71%)	0.478^#^
M3	49 (82%)	77 (82%)	1.000^#^
M4	17 (28%)	24 (26%)	0.852^#^
M5	5 (8%)	5 (5%)	0.515^#^
L1	3 (5%)	8 (9%)	0.529^#^
L2	7 (12%)	9 (10%)	0.789^#^
L3	2 (3%)	4 (4%)	1.000^#^
L4	2 (3%)	1 (1%)	0.561^#^
W1	17 (28%)	19 (20%)	0.054^#^
W2	41 (68%)	60 (65%)	
W3	2 (3%)	14 (15%)	
Defect length (cm)	7 (5–10)	8 (5–12)	0.146*
Defect width (cm)	5 (4–7)	5 (4–8)	0.354*
Defect Size (cm^2^)	36 (21–60)	42 (24–79)	0.119*
Combined M- and L-hernia Defect	6 (10%)	13 (14%)	0.617^#^

^*^Mann–Whitney *U*-test; ^#^chi-squared test/Fisher’s exact test

**Table 2:** Learning curve of eTEP cases, perioperative data

	First 60 eTEP-RS cases (*n* = 60)	eTEP-RS cases 61–153 (*n* = 93)	*p*-value
*Chemical component separation*	0 (0%)	2 (2%)	0.520^#^
Access			
Laparoscopic	59 (98%)	56 (60%)	< 0.001^#^
Robotic-assisted	1 (2%)	37 (40%)	
Conversion	2 (3%)	1 (1%)	
Posterior component separation (transversus abdominis release)			
No	38 (63%)	73 (79%)	0.012^#^
One side	16 (27%)	19 (21%)	
Bilateral	6 (10%)	1 (1%)	
Operation time (min)	189 (138–243)	165 (126–195)	0.029*
Drain placement	36 (60%)	67 (72%)	0.158^#^
No. of Drains—mesh	1 (0–1)	1 (0–1)	0.941*
Days with any drain	2 (0–5)	3 (0–4)	0.667*
Mesh type			
Prolene soft®	39 (60%)	62 (67%)	< 0.001^#^
Ultra Pro®	21 (35%)	6 (7%)	
DynaMesh CICAT®	0 (0%)	25 (27%)	
Mesh—length (cm)	25 (20–30)	27 (22–30)	0.160*
Mesh—width (cm)	15 (12–20)	15(13–16)	0.162*
Mesh size (cm^2^)	375 (299–497)	364 (318–500)	0.993*
Mesh-to-defect ratio	10.8 (7.5–18.8)	8.8 (5.0–14.2)	0.014*
Mesh fixation	6 (10%)	0 (0%)	0.003^#^
NRS-11 pain			
Day 1—rest	1 (0–3)	2 (2–4)	0.012*
Day 1—max	2 (0–4)	3 (2–4)	0.002*
Day 2—rest	1 (0–2)	1 (0–2)	0.599*
Day 2—max	2 (0–4)	2 (0–3)	0.384*
Day 3—rest	0 (0–2)	1 (0–2)	0.237*
Day 3—max	0 (0–2)	1 (0–3)	0.076*
Discharge—rest	0 (0–0)	0 (0–1)	0.015*
Discharge—max	0 (0–0)	0 (0–2)	0.003*
Peridural catheter	4 (7%)	3 (3%)	0.434^#^
Days with PDK	0 (0–0)	0 (0–0)	0.553*
Opiods at discharge	26 (43%)	42 (45%)	0.869^#^
Time to first bowel movement (TTFBM) (days)	3 (2–3)	2 (2–2)	< 0.001*
Length of Stay (days)	6 (4–8)	4 (3–5)	< 0.001*
Length of ICU/IMC-stay (days)	0 (0–1)	0 (0–0)	0.003*
ICU readmission	8 (14%)	0 (0%)	< 0.001^#^
Clavien–Dindo classification			
Grade 0	49 (82%)	79 (85%)	0.041^#^
Grade 1	1 (2%)	7 (8%)	
Grade 2	1 (2%)	4 (4%)	
Grade 3a	0 (0%)	0 (0%)	
Grade 3b	8 (13%)	3 (3%)	
Grade 4a	1 (2%)	0 (0%)	
Grade 4b	0 (0%)	0 (0%)	
Grade 5	0 (0%)	0 (0%)	
Clavien–Dindo classification			
Grade 0–3a	51 (85%)	90 (97%)	0.012^#^
≥ Grade 3b	9 (15%)	3 (3%)	
Re-Operation	9 (15%)	3 (3%)	0.012^#^
Seroma (req. intervention)	1 (2%)	0 (0%)	0.397^#^
Hematoma (req. intervention)	5 (8%)	1 (1%)	0.037^#^
Wound infection (req. intervention)	1 (2%)	0 (0%)	0.397^#^
Mesh infection (req. intervention)	1 (2%)	0 (0%)	0.397^#^
Blood transfusion	3 (5%)	3 (3%)	0.581^#^
SSOPI	9 (15%)	3 (3%)	0.013^#^
Re-admission (30 days)	2 (3%)	4 (4%)	0.763^#^

^*^Mann–Whitney *U*-*t*est; ^#^chi-squared test/Fisher’s exact test

**Table 3:** Study cohort, Open-RS versus eTEP-RS steady-state cohort

	Unmatched cohorts			Propensity score matching		
	Open-RS cohort (*n* = 154)	eTEP-RS cohort (case 60–153) (*n* = 93)	*p*-value	Open-RS cohort (*n* = 83)	eTEP-RS cohort (case 60–153) (*n* = 83)	*p*-value
*Age*	61 (53–69)	63 (50–72)	0.673*	59 (53–67)	63 (50–72)	0.479*
Sex						
Male	86 (56%)	55 (59%)	0.691#	50 (60%)	49 (59%)	0.874#
Female	68 (44%)	38 (41%)		33 (40%)	34 (41%)	
*BMI*	27.7 (24.5–31.8)	29.0 (24.7–32.4)	0.564*	28.4 (25.2–33.1)	29.4 (24.8–32.5)	0.970*
RCS Charlson score						
0	59 (39%)	26 (28%)	0.358#	25 (30%)	26 (31%)	0.516#
1	45 (29%)	34 (37%)		23 (28%)	29 (35%)	
2	35 (23%)	22 (24%)		25 (30%)	17 (21%)	
≥ 3	14 (9%)	11 (12%)		10 (12%)	11 (13%)	
ASA classification						
ASA 1	5 (3%)	1 (1%)	0.240#	1 (1%)	1 (1%)	0.587#
ASA 2	53 (35%)	26 (28%)		29 (35%)	24 (29%)	
ASA 3	90 (59%)	25 (70%)		50 (60%)	57 (69%)	
ASA 4	5 (3%)	1 (1%)		3 (4%)	1 (1%)	
Smoking history						
Yes	46 (30%)	32 (35%)	0.480#	28 (34%)	28 (34%)	1.000#
Anticoagulation						
Yes	20 (13%)	13 (14%)	0.849#	13 (16%)	13 (16%)	1.000#
Type of hernia						
Primary ventral hernia	25 (16%)	20 (21%)	0.312#	16 (19%)	18 (22%)	0.701#
Incisional hernia	129 (84%)	73 (79%)		67 (81%)	65 (78%)	
*Recurrent hernia*	24 (16%)	10 (11%)	0.343#	12 (15%)	9 (11%)	0.484#
EHS classification						
M1	18 (12%)	22(24%)	0.020#	15 (18%)	14 (17%)	0.838#
M2	94 (61%)	66 (71%)	0.131#	54 (65%)	56 (68%)	0.743#
M3	113 (73%)	77 (82%)	0.119#	68 (82%)	68 (82%)	1.000#
M4	42 (27%)	24 (26%)	0.882#	28 (34%)	23 (28%)	0.400#
M5	6 (4%)	5 (5%)	0.752#	4 (5%)	5 (6%)	1.000#
L1	5 (3%)	8 (9%)	0.082#	4 (5%)	5 (6%)	1.000#
L2	17 (11%)	9 (10%)	0.832#	6 (7%)	8 (10%)	0.576#
L3	8 (5%)	4 (4%)	1.000#	3 (4%)	3 (4%)	1.000#
L4	1 (1%)	1 (1%)	1.000#	0 (0%)	1 (1%)	1.000#
W1	26 (17%)	19 (20%)	0.009#	11 (13%)	18 (22%)	0.060#
W2	121 (79%)	60 (65%)		68 (82%)	55 (66%)	
W3	7 (5%)	14 (15%)		4 (5%)	10 (12%)	
Defect length (cm)	7 (5–10)	8 (5–12)	0.134*	7 (5–12)	7 (5–12)	0.597*
Defect width (cm)	5 (4–7)	5 (4–8)	0.970*	6 (4–7)	5 (4–8)	0.355*
Defect size (cm^2^)	40 (24–67)	42 (24–79)	0.251*	49 (25–81)	40 (18–75)	0.587*
Combined M- and L-hernia Defect	9 (6%)	13 (14%)	0.038#	7 (8%)	10 (12%)	0.442#

^*^Mann–Whitney *U*-test; ^#^chi-squared test/Fisher’s exact test

**Table 4:** Perioperative parameters

	Unmatched cohorts			Propensity score matching		
	Open-RS cohort (*n* = 154)	eTEP-RS cohort (case 60–153) (*n* = 93)	*p*-value	Open-RS cohort (*n* = 83)	eTEP-RS cohort (case 60–153) (*n* = 83)	*p*-value
Prior mesh	9 (6%)	4 (4%)	0.772^#^	5 (6%)	4 (5%)	1.000^#^
Mesh removal	1 (1%)	0 (0%)	1.000^#^	1 (0%)	0 (0%)	1.000^#^
Chemical component separation	0 (0%)	2 (2%)	0.141^#^	0 (0%)	2 (2%)	0.497^#^
Access						
Open	154 (100%)	0 (0%)	< 0.001^#^	83 (100%)	0 (0%)	< 0.001^#^
Laparoscopic	0 (0%)	56 (60%)		0 (0%)	53 (64%)	
Robotic-assisted	0 (0%)	37 (40%)		0 (0%)	30 (36%)	
Conversion	N.A	1 (1%)		N.A	1 (1%)	
eTEP—crossover						
Superior	N.A	47 (51%)	N.A	N.A	44 (54%)	N.A
Inferior	N.A	38 (41%)		N.A	32 (39%)	
N.A	N.A	8 (9%)		N.A	6 (7%)	
Posterior component Separation (transversus abdominis release)						
No	131 (85%)	73 (79%)	0.119^#^	73 (88%)	68 (82%)	0.100^#^
One-side	17 (11%)	19 (21%)		6 (7%)	14 (17%)	
Bilateral	6 (4%)	1 (1%)		4 (5%)	1 (1%)	
Operation time (min)	125 (94–159)	165 (126–195)	< 0.001*	135 (95–161)	160 (126–192)	0.004*
Drain placement	149 (97%)	67 (72%)	< 0.001^#^	80 (96%)	60 (72%)	< 0.001^#^
No. of drains—mesh	2 (1.75–2)	1 (0–1)	< 0.001*	2 (2–2)	1 (0–1)	< 0.001*
No. of drains—subcutaneous	1 (1–2)	0 (0–0)	< 0.001*	1 (1–2)	0 (0–0)	< 0.001*
Days with any drain	5 (4–6)	3 (0–4)	< 0.001*	5 (4–6)	3 (0–4)	< 0.001*
Cumulative drain volume (ml) (if drain was placed)	390 (235–653)	300 (180–500)	< 0.016*	420 (267–708)	302 (180–500)	< 0.010*
Mesh type						
Prolene Soft®	5 (3%)	62 (67%)	< 0.001^#^	4 (5%)	52 (63%)	< 0.001^#^
Ultra Pro®	148 (96%)	6 (7%)		79 (95%)	6 (7%)	
DynaMesh CICAT®	0 (0%)	25 (27%)		0 (0%)	25 (30%)	
Symbotex®	1 (1%)	0 (0%)		0 (0%)	0 (0%)	
Mesh—length (cm)	18 (15–27)	27 (22–30)	< 0.001*	22 (15–30)	27 (22–30)	< 0.001*
Mesh—width (cm)	15 (13–17)	15(13–16)	0.185*	15 (14–18)	15 (13–15)	0.054*
Mesh size (cm^2^)	325 (225–468)	364 (318–500)	0.006*	330 (225–468)	360 (286–493)	0.125*
Mesh-to-defect-ratio	7.7 (5.0–13.5)	8.8 (5.0–14.2)	0.660*	7.4 (4.8–13)	9.1 (5.2–15.2)	0.177*
Mesh fixation	151 (98%)	0 (0%)	< 0.001^#^	81 (98%)	0 (0%)	< 0.001^#^
NRS-11-pain						
Day 1—rest	2 (0–4)	2 (2–4)	0.884*	2 (0–4)	2 (1–4)	0.619*
Day 1—max	3 (1–5)	3 (2–4)	0.342*	3 (1–5)	3 (2–5)	0.221*
Day 2—rest	2 (0–3)	1 (0–2)	0.117*	2 (0–4)	1 (0–2)	0.034*
Day 2—max	2.5 (0–5)	2 (0–3)	0.068*	3 (0–5)	2 (0–3)	0.015*
Day 3—rest	1 (0–3)	1 (0–2)	0.223*	1 (0–3)	0 (0–2)	0.043*
Day 3—max	2 (0–4)	1 (0–3)	0.114*	2 (0–4)	1 (0–3)	0.076*
Discharge—rest	0 (0–1)	0 (0–1)	0.313*	0 (0–1)	0 (0–1)	0.412*
Discharge—max	0 (0–1)	0 (0–2)	0.095*	0 (0–2)	0 (0–2)	0.337*
Peridural catheter	43 (28%)	3 (3%)	< 0.001^#^	20 (25%)	2 (2%)	< 0.001^#^
Days with peridural catheter	0 (0–2)	0 (0–0)	< 0.001*	0 (0–1)	0 (0–0)	< 0.001*
Opiods at discharge	62 (41%)	42 (45%)	0.502^#^	31 (38%)	38 (46%)	0.330^#^
Time to first bowel movement (days)	2 (2–3)	2 (2–2)	0.005*	2 (2–3)	2 (2–2)	0.016*
Length of stay (days)	7 (5–9)	4 (3–5)	< 0.001*	7 (6–9)	4 (3–5)	< 0.001*
Length of ICU/IMC-stay (days)	0 (0–2)	0 (0–0)	< 0.001*	0 (0–2)	0 (0–0)	0.004*
ICU readmission	14 (9%)	0 (0%)	0.004^#^	8 (10%)	0 (0%)	0.003^#^
Clavien–Dindo classification						
Grade 0	106 (69%)	79 (85%)	0.146^#^	55 (66%)	70 (84%)	0.064^#^
Grade 1	16 (11%)	7 (8%)		10 (12%)	7 (8%)	
Grade 2	13 (9%)	4 (4%)		7 (8%)	3 (4%)	
Grade 3a	5 (3%)	0 (0%)		3 (4%)	0 (0%)	
Grade 3b	7 (5%)	3 (3%)		3 (4%)	3 (4%)	
Grade 4a	3 (2%)	0 (0%)		3 (4%)	0 (0%)	
Grade 4b	2 (1%)	0 (0%)		2 (2%)	0 (0%)	
Grade 5	1 (1%)	0 (0%)		0 (0%)	0 (0%)	
Clavien–Dindo classification						
Grade 0-3a	140 (91%)	90 (97%)	0.118^#^	75 (90%)	80 (96%)	0.131^#^
≥ Grade 3b	13 (9%)	3 (3%)		8 (10%)	3 (4%)	
Re-operation	9 (6%)	3 (3%)	0.543^#^	4 (5%)	3 (4%)	1.000^#^
Seroma (req. intervention)	5 (3%)	0 (0%)	0.160^#^	4 (5%)	0 (0%)	0.043^#^
Hematoma (req. Intervention)	3 (2%)	1 (1%)	1.000^#^	2 (3%)	1 (1%)	1.000^#^
wound infection (req. Intervention)	6 (4%)	0 (0%)	0.086^#^	3 (4%)	0 (0%)	0.245^#^
Mesh infection (req. Intervention)	4 (3%)	0 (0%)	0.300^#^	3 (4%)	0 (0%)	0.245^#^
Blood transfusion	8 (5%)	3 (3%)	0.467^#^	5 (6%)	3 (4%)	0.468^#^
SSOPI	15 (10%)	3 (3%)	0.075^#^	8 (10%)	3 (4%)	0.131^#^
Re-admission (30 days)	6 (4%)	4 (4%)	0.288^#^	6 (7%)	3 (4%)	0.514^#^

^*^Mann–Whitney *U*-test; ^#^chi-squared test/Fisher’s exact test

### Learning curve of eTEP-RS

**Fig. 3:**
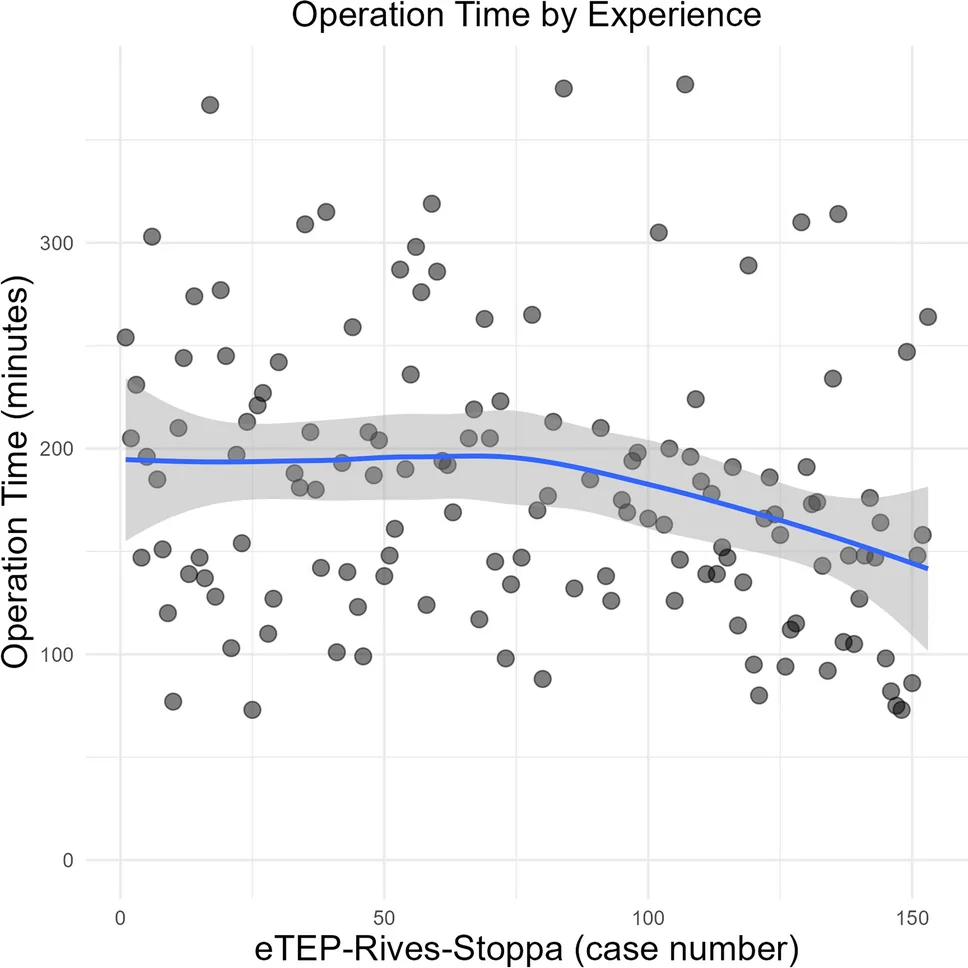
Learning curve of eTEP-RS: A reduction of the mean operation time can be seen after approximately 60 eTEP-RS cases (all eTEP-RS cases: *n* = 153)

While evaluating the data, we found that after approximately 60 eTEP-RS cases performed at our center, a distinct reduction of the necessary operation times was observed, indicating a relevant learning curve for eTEP-RS procedures (Fig. 3, Fig. S1A).

This observation led to a subgroup analysis of the eTEP-RS cases. Table 1 demonstrates the demographics of the eTEP-RS learning cohort (cases 1–60) and the eTEP-RS steady-state cohort (cases 61–153). No significant differences in the demographic data could be observed in the two periods, while a trend toward slightly larger hernias could be observed in the eTEP-RS steady-state cohort.

Significant differences in the results were observed when analyzing the perioperative data of these two cohorts (Table 2). Median operation time was significantly reduced by 24 min in the eTEP-RS steady-state cohort. Posterior component separation was used less frequently despite more combined lateral and medial hernias and a trend toward larger hernia defects. This may be due to better preparation with higher rate of posterior sheath preservation even in incisional hernia cases and a more frequent use of hernia sac-bridging of the posterior layer in cases where TAR for posterior layer closure would have been necessary in the learning cohort. Furthermore, during the learning curve of the eTEP-RS procedure, the complication rate was significantly higher. Complications during the learning curve were mainly retrorectus hematoma (*n* = 4) and posterior sheath-associated problems (*n* = 2), which were all managed by minimally invasive revision. Three patients in the eTEP-RS learning cohort needed open revisional surgery. One patient with severe arteriosclerosis developed postoperative colonic ischemia without abdominal compartment syndrome or local complications after eTEP-RS (open revision with mesh explantation and colonic resection). One patient developed a subcutaneous burst abdomen, which was treated by open surgical revision with reposition of the mesh and open fascial reconstruction. One patient developed a low-output enteric fistula to the retromuscular space after laparoscopic adhesiolysis and prior revision due to hematoma. This patient underwent open revision on the 19th postoperative day with mesh explantation and bowel resection. This patient also is one of three patients with hernia recurrence in the eTEP-RS cohort and underwent open Rives–Stoppa repair after 10 months with an uneventful postoperative course.

In the eTEP-RS cohort, significantly fewer perioperative complications were observed in 93 consecutive patients. Only one patient with therapeutic anticoagulation with postoperative hematoma, one patient with posterior sheath defect and interparietal hernia, and one patient with ileus due to a minor tear in the posterior layer needed revisional surgery (3%). All these cases were managed by minimally invasive re-operation.

### Perioperative data

To address the problem of learning curve contamination of the primary analysis of this manuscript, we decided to only match the eTEP-RS steady-state cohort (*n* = 93) to the Open-RS cohort (which was performed in the steady-state phase of this procedure at our clinic) to be able to analyze the true effects of eTEP-RS on perioperative and long-term outcomes compared to Open-RS procedures.

The demographic data and perioperative data of the unmatched and propensity score-matched cohorts are shown in Tables 3 and 4.

After propensity score matching, 83 patients were available in each group. The groups were well balanced with regard to age, BMI, comorbidities, as well as hernia-related characteristics after propensity score matching, without significant differences observed between groups. With regard to perioperative comparative efficacy of eTEP-RS compared to Open-RS, several statistically significant differences were observed between the two procedures (Table 4): Median operation time was 25 min longer in the eTEP-RS cohort. Posterior component separation by transversus abdominis release was used non-significantly more often after eTEP-RS.

Interestingly, the operation time was correlated with the defect size (cm^2^) in the eTEP-RS steady-state cohort (Pearson correlation coefficient: 0.637; *p* < 0.001) but not in the matched Open-RS cohort (Pearson correlation coefficient: 0.152; *p* = 0.07; Fig. S1B). The same observation was also made for defect width (eTEP-RS steady-state cohort: Pearson correlation coefficient: 0.491; *p* < 0.001; matched Open-RS cohort: Pearson correlation coefficient: 0.022; *p* = 0.8; Fig. S1C). This indicates that greater defect size is correlated with a more complex surgical procedure in eTEP-RS cases, but not in Open-RS cases of similarly sized defects in the selected cohort.

While we changed the mesh products used over time, which is reflected in our data, median mesh size in cm^2^ and mesh-to-defect ratio (MDR) did not differ significantly between eTEP-RS and Open-RS procedures. Penetrating mesh fixation (transfascial sutures used in Open-RS) and drain placement was used less frequently in the eTEP-RS cohort.

When comparing postoperative recovery after both procedures, necessity for and length of postoperative ICU observation was shorter in the eTEP-RS steady-state cohort, and overall length of hospital stay was shorter in the eTEP-RS steady-state cohort. Peridural catheters were placed more frequently in the Open-RS cohort. Despite this more invasive postoperative pain treatment, NRS-11 pain scores were lower in the eTEP-RS steady-state cohort on postoperative days 2–3, indicating faster recovery from surgery. The frequency of hospital discharge with oral opioids, however, did not differ between the two cohorts.

Regarding perioperative complications, while not significant, a clear trend toward fewer complications, fewer SSOPI events, and fewer re-admissions can be observed following eTEP-RS procedures compared to Open-RS procedures. There were no mortalities after eTEP-RS repair, while one patient died after Open-RS due to acute pulmonary embolism.

### Management of posterior sheath defects in eTEP-RS cases

**Fig. 4:**
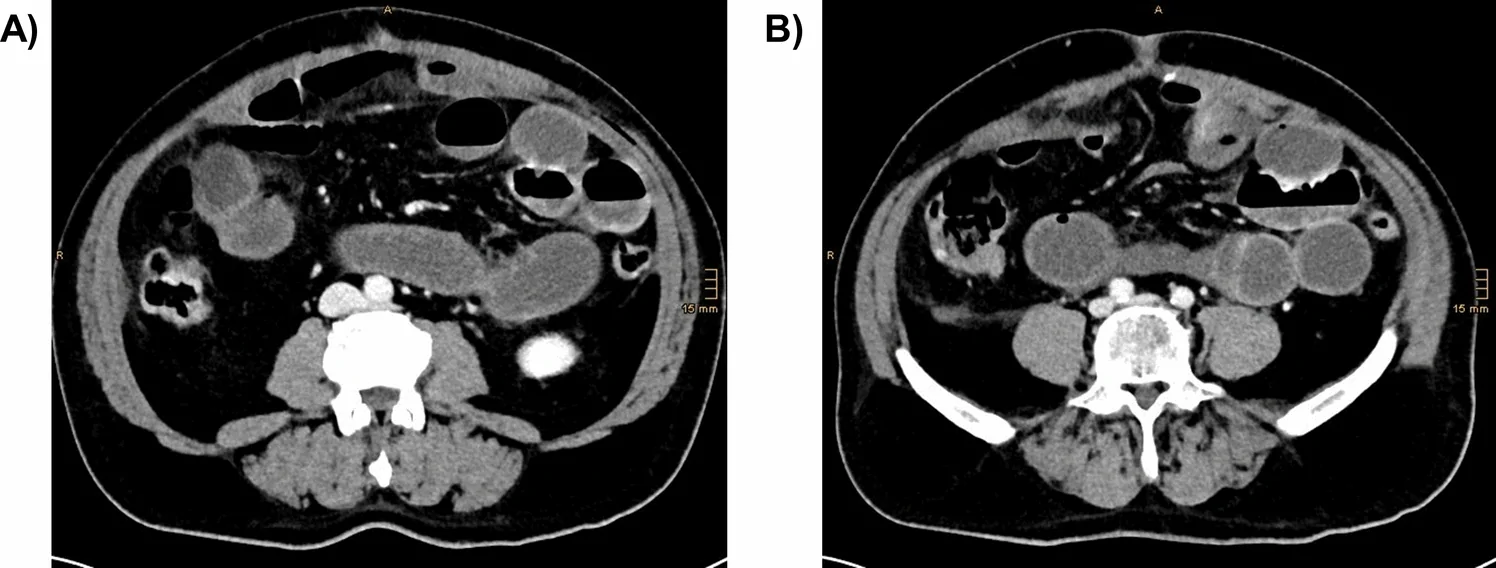
**A** and **B** Example CT scan of an interparietal hernia due to a posterior sheath dehiscence after eTEP Rives–Stoppa repair. Emergency CT scan upon suspicion is indicated with immediate surgical revision (usually laparoscopic management will be possible) due to possible incarceration of bowel

In our entire eTEP-RS cohort of 153 patients, 4 patients (3%) underwent minimally invasive re-operation due to posterior sheath defects. Only two of these were true posterior sheath disruptions with larger interparietal hernias (Fig. 4), and two were patients with postoperative mechanical ileus due to small tears in the posterior sheath with bowel adhesions to the mesh or barbed suture material. An urgent CT scan is necessary if interparietal hernia is suspected. A true interparietal hernia should be managed by peracute surgical intervention due to the risk of incarceration, while mechanical ileus without risk of incarceration can be treated according to routine emergency management of mechanical ileus. These defects were all managed by minimally invasive trans-abdominal revision without mesh removal and closure of the posterior layer by bridging the defect with an absorbable mesh (Polyglactin, Vicryl, Ethicon, Raritan, New Jersey, USA).

### Recurrence and long-term quality of life

**Fig. 5:**
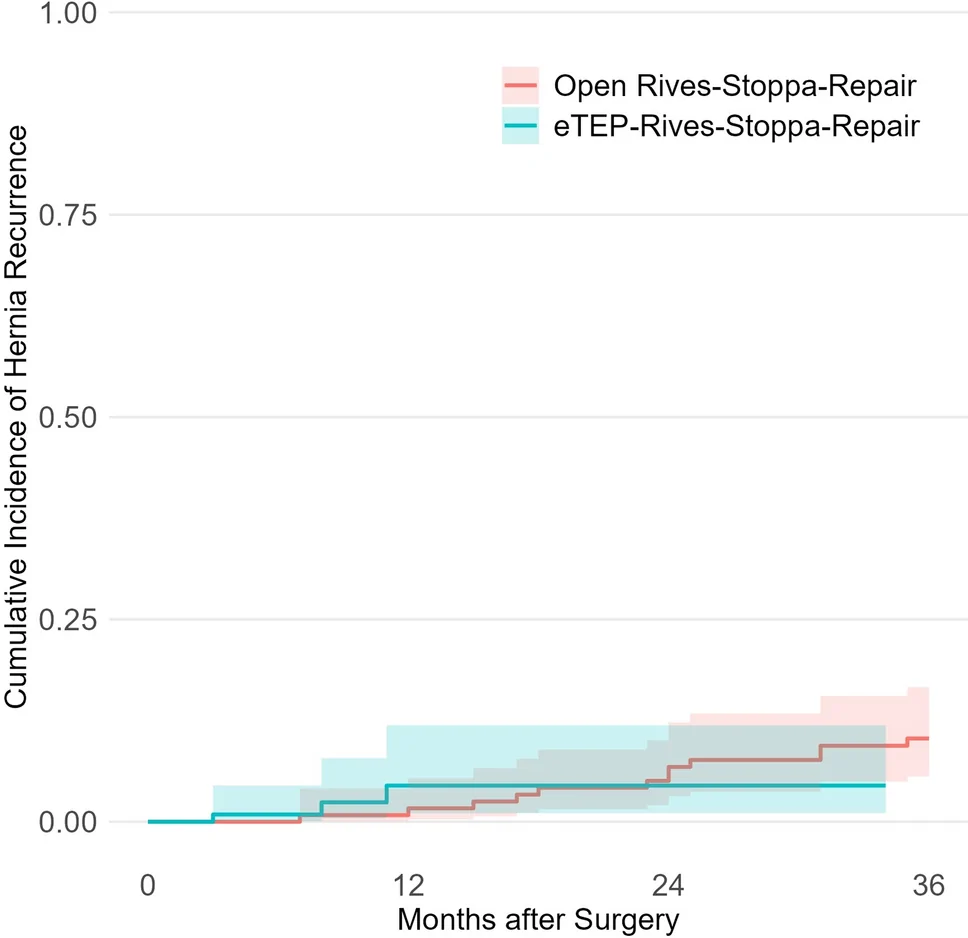
Hernia recurrence rates after Open Rives–Stoppa repair and eTEP Rives–Stoppa repair. Aalen–Johansen estimator for competing risk analysis and Fine–Grey analysis: There is no statistically significant difference in hernia recurrencerates between both approaches (HR 0.83 (95% confidence Interval (CI): 0.26–2.64, *p* = 0.76). 1-year recurrence rate was 2% (95%-CI 0–6%) after Open Rives–Stoppa repair and 5% (95%-CI 1–12%, *p* > 0.9) after eTEP Rives–Stoppa repair. 2-year recurrence rate was 7% (95%-CI: 3–12%) after Open Rives–Stoppa repair and 5% (95%-CI 1–12%, *p* > 0.9) after eTEP Rives–Stoppa repair. Longer follow-up intervals in the eTEP-RS cohort are needed

The competing risk analysis showed no statistically significant difference in hernia recurrence rates between the two approaches (HR 0.83 (95% confidence interval (CI) 0.26–2.64, *p* = 0.76). 1-year recurrence rate was 2% (95% CI 0–6%) after Open Rives–Stoppa repair and 5% (95% CI 1–12%, *p* > 0.9) after eTEP Rives–Stoppa repair. The 2-year recurrence rate was 7% (95% CI 3–12%) after Open Rives–Stoppa repair and 5% (95% CI 1–12%, *p* > 0.9) after eTEP Rives–Stoppa repair. Longer follow-up intervals in the eTEP-RS cohort are needed in a future long-term observational study of the cohort (Table 5, Fig. 5).

**Table 5:** Grading of hernia recurrence events according to Lima et al.

Recurrence grade	Open-RS cohort (*n* = 154%)	eTEP-RS cohort (*n* = 153%)
Grade 0—no recurrence	133 (86)	150 (98)
Grade Ia—asymptomatic (Identified by imaging, no need for intervention)	3 (2)	0 (0)
Grade Ib—asymptomatic (Patient reported, no need for intervention)	5 (3)	1 (1)
Grade IIa—symptomatic (Watchful waiting)	5 (3)	1 (1)
Grade IIb—symptomatic—requires elective repair	3 (2)	1 (1)
Grade III—urgent repair	3 (2)	0 (0)
Grade IV—life-threatening complications related to recurrence	2 (1)	0 (0)
Grade V—death due to recurrence	0 (0)	0 (0)
Grade VI—death of patient not related to recurrence	0 (0)	0 (0)

With regard to long-term quality of life, we performed an exploratory and hypothesis-generating analysis with 84 (55%) patients of the Open-RS cohort and 138 (90%) patients of the eTEP-RS cohort, who answered the Carolina Comfort Scale questionnaire at a median follow-up of 58 months (Open-RS cohort) and 7 months (eTEP-RS cohort). Patients were more satisfied with the eTEP procedure in general, while overall Carolina Comfort Scale scores were not different between cohorts. The Carolina Comfort Scale pain score was better for eTEP-RS, while the Carolina Comfort Scale mesh sensation score was better for Open-RS. No differences regarding movement limitations were observed (Table S4). These results have to be interpreted in view of the different follow-up intervals of the cohorts.

## Discussion

In this manuscript, we present the largest cohort study comparing eTEP Rives–Stoppa repair and Open Rives–Stoppa repair for patients undergoing ventral hernia surgery. eTEP-RS is a safe procedure compared to Open-RS and appears to offer favorable outcomes regarding short-term perioperative aspects, such as reduced postoperative pain, ICU, and hospital length of stay due to faster recovery of patients from the surgical procedure.

With regard to long-term recurrence after eTEP-RS, we did not observe significant differences regarding recurrence rates. As a potential confounder within recurrence and quality-of-life analyses, we did change the used mesh products over time due to concerns with the previously used lightweight mesh material in ventral hernia repair of medium-to-large-sized hernias [59]. With regard to recurrence, we observed three recurrences after eTEP-RS, of which one patient needed re-operation with mesh removal due to small bowel fistula after laparoscopic adhesiolysis and eTEP-RS repair. Recurrence in that case must be considered as a direct consequence following a surgical complication. One patient had an asymptomatic partial recurrent diastasis after diastasis plication and umbilical hernia treatment with eTEP-RS and one patient had a small recurrent hernia at the lateral edge of a Makuuchi incision after eTEP-RS with right-sided TAR. These observations have to be viewed in light of short follow-up intervals in the eTEP cohort and underpowered sample sizes for non-inferiority analysis. Therefore, longer follow-up intervals and larger (multicenter) cohorts will be necessary to provide more robust data in future.

Long-term quality of life was not different between the two procedures in the total Carolina Comfort Scale score, while eTEP-RS was superior with regard to pain and Open-RS was superior with regard to mesh sensation. This analysis is exploratory and hypothesis-generating with severely limited generalizability as the different follow-up intervals due to the study design and the different mesh implants used may have influenced the findings. Furthermore, due to the retrospective design of the study, preoperative Carolina Comfort Scale data were not available for all patients, which further limits the evaluability of the data.

The main disadvantage of eTEP-RS was the prolonged operation time compared to Open-RS procedures, which led to a greater consumption of hospital resources (operating room time, anaesthesiologist time, surgeon workload). In accordance with a new technique´s learning curve, we could demonstrate shorter operation times with increasing case numbers. After completion of the initial learning curve, the additional operation time needed for eTEP-RS was reduced to 25 min in this study between eTEP-RS and Open-RS. Despite the clear clinical advantages of eTEP-RS for suitable patients, further health economic evaluation of eTEP-RS versus Open-RS will be necessary to evaluate whether the additional use of OR resources is outweighed by cost savings for the health care provider/hospital (budget-impact analysis) during the postoperative management of the patients and the general cost-effectiveness analysis of both procedures.

An important additional finding of our study is the relevance of the learning curve of eTEP-RS procedures. As reported by other authors, eTEP-RS has a significant learning curve of at least 30–50 cases for a single surgeon [60–63]. Our data clearly show the decreasing operation times and reduced perioperative complications after approximately 60 cases in the two-surgeon team approach with still ongoing improvements of operation times after 150 cases as demonstrated in Fig. 3. Safe implementation of a novel technique in a surgical unit is of utmost importance and eTEP-RS as a complex minimally invasive abdominal wall procedure should be limited to small, specialized teams (2–3 surgeons with adequate caseloads) within a surgical clinic. With more widespread availability of eTEP-RS performed in many centers, surgeons aiming to newly implement the procedure in their hospitals should use the possibilities of mentoring and cadaver courses prior to implementation of eTEP-RS and eTEP-TAR as a novel approach [63].

The results of our study are in line with previous smaller cohorts comparing the two approaches for retromuscular repair. A Belgian retrospective cohort published by Kinet et al. [21] compared 30 vs 30 patients undergoing eTEP-RS and Open-RS for small ventral hernia (median defect width: 18 mm) with concomitant diastasis and found reduced length of stay and longer operation time for the former [21]. A Spanish retrospective cohort study published by González De Godos et al. in 2025 consisting of 46 Open-RS cases and 80 eTEP-RS cases found lower postoperative pain and longer operation times (127 vs. 255/227 min) for eTEP-RS. Moreover, larger meshes were placed in the eTEP-RS cohort, which supports our findings. Differences regarding hospital stay, complications, and hernia recurrence were not observed. The study has its limitations, however, due to unbalanced cohorts, as the hernia defects treated by minimally invasive approaches were considerably smaller than in the Open-RS cohort and incisional hernias were less frequent in the eTEP-RS cohort [22]. Lastly, a German retrospective cohort study was published by Looft et al. in 2025. 92 patients undergoing eTEP-RS were compared to 47 undergoing Open-RS. The study has limitations regarding generalizability as hernia defects were very small (Median defect size of 6 cm^2^ in the eTEP-RS cohort) and differed significantly between the cohorts. Only primary hernias (umbilical hernia) were treated and no incisional hernia cases were included. However, a reduction in length of stay and particularly in perioperative complications was observed. This was due to significantly fewer minor complications (Grade I and II) by local wound complications in the eTEP-RS cohort. This result is expected and appears to be compatible with fundamental advantages of laparoscopic surgery compared to open approaches [20].

Compared to those prior studies, the advantages of our study are larger and well-balanced/matched cohorts with a relatively high number of incisional hernias and reasonable defect sizes. Still, we can see similar effects in terms of general complication rate and length of stay in our eTEP-RS cohort, especially after completion of the learning curve.

The length of stay after eTEP-RS was significantly shorter compared to Open-RS in our cohort, but still quite long compared to other eTEP-RS studies [19]. This may be explained by patients with high grades of comorbidity at our university medical center on the one hand and by fundamental accompanying factors of the German healthcare system on the other hand. Reduction of hospital length of stay is a current political aim in Germany [64], but in the meantime, our length of stay following eTEP-RS is within the reports of other German authors [65, 66].

Evaluating the comparative effectiveness between Open-RS and eTEP-RS is of clinical relevance, as the rates of laparoscopic IPOM have been steadily decreasing during the past years while the rates of patients being treated by Open-RS are increasing [67]. Only a small portion of patients is treated by eTEP-RS or comparable minimally invasive extraperitoneal approaches. Further widespread (but safe) implementation of eTEP-RS could improve patient outcomes, so that patients can profit simultaneously from minimally invasive approaches and the extraperitoneal mesh placement.

From our point of view, eTEP-RS is the best approach for medium-sized and in selected cases large ventral hernia, if the procedure can technically be performed in the given patient and hernia characteristics. Otherwise, Open-RS still has its place for large or otherwise complicated ventral hernia repair. Others may opt for IPOM repair, and short-term results may be similar between those procedures [19, 68]. Whether adverse long-term consequences of intraperitoneal mesh placement in IPOM repairs can be avoided by eTEP-RS will be shown in future long-term follow-up studies. Regarding the recommendations of the latest guideline for incisional hernia treatment, extraperitoneal mesh placement should be favored in the repair of medium-sized ventral hernias [9]. Whether perioperative results for patients will be improved and surgeon workload will be reduced by the increasing application of robotic-assisted eTEP-RS procedures is subject to ongoing research and technical innovation [15, 33, 69–71].

Limitations of our current study include the retrospective and longitudinal design of the study. While control cases were retrospectively reviewed for eTEP feasibility to minimize bias, and the cohorts were well matched and comparable, other factors cannot be controlled in this study design and may induce bias. A randomized trial comparing the two approaches would, of course, be the better way to generate robust evidence. In our opinion, the advantages of eTEP-RS for suitable patients are distinct and it is unclear whether such a (multicenter) trial would be feasible due to patients and surgeons not willing to randomize participants. Other limitations may be due to differences in perioperative management (differences in drain usage, peridural anesthesia). We acknowledge that some portion of the observed short-term benefits of eTEP-RS, such as pain and length-of-stay advantages, may be to some extent be attributable to those differences in perioperative management rather than the minimally invasive approach per se. Although no funding for this study was received, several authors of this manuscript were supported by Intuitive Surgical in the past as stated in the disclosures of this study. This may induce bias as eTEP-RS is a procedure that gained widespread attention for its suitability for robotic surgery. Another limitation of our cohort is the rather short follow-up interval in the eTEP-RS cohort, which warrants future long-term follow-up of this patient cohort within a separate long-term follow-up cohort study.

## Conclusion

eTEP Rives–Stoppa repair offered superior short-term outcomes and comparable long-term recurrence rates and health-related quality of life compared to open Rives–Stoppa repair in suitable patients with medium-sized and in selected cases large ventral hernia. These advantages for the patients came at the cost of longer operation times and therefore higher utilization of hospital resources. Future studies with larger cohorts with longer follow-up periods are necessary in future.

## Supplementary Information

Below is the link to the electronic supplementary material.

Supplementary file1 (DOCX 299 KB)

## Data Availability

The datasets generated and analyzed during the current study are available from the corresponding author upon reasonable request.
