# Supplementary material for: Enhanced view/extended totally extraperitoneal plasty (eTEP) Rives–Stoppa repair versus open Rives–Stoppa repair: a single-center retrospective propensity score-matched cohort study
Source: Surg Endosc. 2026 Jul 2;40(9):7548–64. doi: 10.1007/s00464-026-13049-0 (PMC13582168; doi:10.1007/s00464-026-13049-0)
Supplement: Supplementary file 1 [file 464_2026_13049_MOESM1_ESM.docx]

**Supplementary Data:** **Enhanced-view/extended totally extraperitoneal plasty (eTEP)-Rives-Stoppa-repair versus open Rives-Stoppa-repair – a single center retrospective propensity score matched cohort study**

Robin Klewitz, Nico Pfander, York Gruenewald, Hans Christian Hillebrecht, Stefan Fichtner-Feigl, Philipp Anton Holzner and Julian Hipp

**Fig. S1:**

A) Reduction of operation time was independent from introduction of robotic-assisted eTEP-procedures at our center.

B) The operation time was correlated with the defect size (cm²) in the eTEP-RS-steady state-cohort (Pearson correlation coefficient: 0.637; p<0.001) while not in the matched Open-RS-cohort (Pearson correlation coefficient: 0.152; p=0.07).

C) The same observation was also made for defect width (eTEP-RS-steady state-cohort: Pearson correlation coefficient: 0.491; p<0.001; Matched Open-RS-cohort: Pearson correlation coefficient: 0.022; p=0.8).

This may indicate, that higher defect size is correlated to a more complex surgical procedure in eTEP-RS-cases, while not in Open-RS-cases of similarly sized defects in the selected cohort.

**Table S1:** Comparison of Open Sublay-Rives-Stoppa-procedures from 01/2018-02/2023 with regard to retrospective eTEP-feasibility-assessment. The „eTEP-not feasible“-cohort had a high rate of W3-hernia, hernia in difficult locations (M1,M5, L2-3) compared to the final study control cohort.

|  | | **eTEP-feasible**  **(n=154)** | **eTEP-not feasible (n=84)** | **eTEP-feasibility could not be assessed**  **(n=71)** |
| --- | --- | --- | --- | --- |
| **Age** | | 61 (53-69) | 65 (54-71) | 61 (51-70) |
| **BMI** | | 27.7 (24.5-31.8) | 28.2 (24.6-32.7) | 27.7 (24.9-31.3) |
| **Type of hernia** | **Primary ventral hernia** | 25 (16%) | 4 (5%) | 15 (21%) |
|  | **Incisional hernia** | 129 (84%) | 80 (95%) | 57 (79%) |
| **Recurrent hernia** | | 24 (16%) | 17 (20%) | 9 (13%) |
| **EHS-classification** | **M1** | 18 (12%) | 14 (17%) | Missing data |
|  | **M2** | 93 (60%) | 41 (49%) | Missing data |
|  | **M3** | 112 (73%) | 43 (51%) | Missing data |
|  | **M4** | 41 (27%) | 35 (42%) | Missing data |
|  | **M5** | 6 (4%) | 12 (14%) | Missing data |
|  | **L1** | 6 (4%) | 8 (10%) | Missing data |
|  | **L2** | 18 (12%) | 15 (18%) | Missing data |
|  | **L3** | 9 (6%) | 12 (14%) | Missing data |
|  | **L4** | 1 (1%) | 2 (2%) | Missing data |
|  | **W1** | 26 (17%) | 4 (5%) | Missing data |
|  | **W2** | 121 (79%) | 30 (40%) | Missing data |
|  | **W3** | 7 (5%) | 41 (55%) | Missing data |
| **Defect length (cm)** | | 7 (5-10) | 10 (7-15) | Missing data |
| **Defect width (cm)** | | 5 (4-7) | 10 (7-14) | Missing data |
| **Defect Size (cm²)** | | 40 (24-67) | 110 (52-205) | Missing data |

**Table S2:** Study cohort, Open-RS versus eTEP-RS, all patients

|  | | | **Open-RS-cohort**  **(n=154)** | **eTEP-RS-cohort**  **(all cases)**  **(n=153)** | **p-value** |
| --- | --- | --- | --- | --- | --- |
| **Age** | | | 61 (53-69) | 63 (54-71) | 0.278* |
| **Sex** | | **Male** | 86 (56%) | 91 (60%) | 0.519# |
|  |  | **Female** | 68 (44%) | 62 (40%) |  |
| **BMI** | | | 27.7 (24.5-31.8) | 28.4 (24.0-32.8) | 0.953* |
| **RCS Charlson Score** | **0** | | 59 (39%) | 43 (28%) | 0.257# |
|  | **1** | | 45 (29%) | 56 (37%) |  |
|  | **2** | | 35 (23%) | 40 (26%) |  |
|  | **≥3** | | 14 (9%) | 14 (9%) |  |
| **ASA-Classification** | **ASA 1** | | 5 (3%) | 2 (1%) | 0.265# |
|  | **ASA 2** | | 53 (35%) | 41 (27%) |  |
|  | **ASA 3** | | 90 (59%) | 105 (69%) |  |
|  | **ASA 4** | | 5 (3%) | 5 (3%) |  |
| **Anticoagulation** | **Yes** | | 20 (13%) | 20 (13%) | 1.000# |
| **Type of hernia** | | **Primary ventral hernia** | 25 (16%) | 31 (20%) | 0.361# |
|  |  | **Incisional hernia** | 129 (84%) | 122 (80%) |  |
| **Recurrent hernia** | | | 24 (16%) | 16 (11%) | 0.182# |
| **EHS-classification** | | **M1** | 18 (12%) | 33 (22%) | 0.020# |
|  |  | **M2** | 94 (61%) | 105 (69%) | 0.164# |
|  |  | **M3** | 113 (73%) | 126 (82%) | 0.058# |
|  |  | **M4** | 42 (27%) | 41 (27%) | 0.925# |
|  |  | **M5** | 6 (4%) | 10 (7%) | 0.298# |
|  |  | **L1** | 5 (3%) | 11 (7%) | 0.120# |
|  |  | **L2** | 17 (11%) | 16 (11%) | 0.869# |
|  |  | **L3** | 8 (5%) | 6 (4%) | 0.593# |
|  |  | **L4** | 1 (1%) | 3 (2%) | 0.311# |
|  |  | **W1** | 26 (17%) | 36 (24%) | 0.031# |
|  |  | **W2** | 121 (79%) | 101 (66%) |  |
|  |  | **W3** | 7 (5%) | 16 (11%) |  |
| **Defect length (cm)** | | | 7 (5-10) | 7 (5-11) | 0.360* |
| **Defect width (cm)** | | | 5 (4-7) | 5 (4-7) | 0.588* |
| **Defect Size (cm²)** | | | 40 (24-67) | 38 (24-75) | 0.719* |
| **Combined M- and L-Hernia Defect** | | | 9 (6%) | 19 (12%) | 0.045# |

*Mann-Whitney-U-Test; #chi-squared-test/Fisher's exact test

**Table S3:** Perioperative data, Open-RS versus eTEP-RS, all patients

|  | | | **Open-RS-cohort**  **(n=154)** | **eTEP-RS-cohort**  **(all cases)**  **(n=153)** | **p-value** |
| --- | --- | --- | --- | --- | --- |
| **Chemical component separation** | | | 0 (0%) | 2 (1%) | 0.155# |
| **Access** | | **Open** | 154 (100%) | 0 (0%) | <0.001# |
|  |  | **Laparoscopic** | 0 (0%) | 115 (75%) |  |
|  |  | **Robotic-assisted** | 0 (0%) | 38 (25%) |  |
|  |  | **Conversion** | N.A. | 3 (2%) |  |
| **Posterior Component Separation (transversus-abdominis-release)** | | **no** | 131 (85%) | 111 (73%) | 0.018# |
|  |  | **One-side** | 17 (11%) | 35 (23%) |  |
|  |  | **Bilateral** | 6 (4%) | 7 (5%) |  |
| **Operation time (min)** | | | 125 (94-159) | 169 (132-212) | <0.001* |
| **Drain placement** | | | 149 (97%) | 103 (67%) | <0.001# |
| **No. of Drains - Mesh** | | | 2 (1.75-2) | 0 (0-1) | <0.001* |
| **No. of Drains – subcutaneous** | | | 1 (1-2) | 0 (0-0) | <0.001* |
| **Days with any drain** | | | 5 (4-6) | 3 (0-5) | <0.001* |
| **Cumulative Drain Volume (ml) (if drain was placed)** | | | 390 (235-653) | 335 (180-570) | 0.289* |
| **Mesh Type** | | **Prolene Soft®** | 5 (3%) | 101 (66%) | <0.001# |
|  |  | **Ultra Pro®** | 148 (96%) | 27 (18%) |  |
|  |  | **DynaMesh CICAT®** | 0 (0%) | 25 (16%) |  |
|  |  | **Symbotex®** | 1 (1%) | 0 (0%) |  |
| **Mesh – Length (cm)** | | | 18 (15-27) | 27 (22-30) | <0.001* |
| **Mesh – Width (cm)** | | | 15 (13-17) | 15 (13-17) | 0.525* |
| **Mesh Size (cm²)** | | | 325 (225-468) | 375 (300-500) | 0.002* |
| **Mesh-to-Defect-Ratio** | | | 7.7 (5.0-13.5) | 9.7 (6.1-16.1) | 0.043* |
| **Mesh fixation** | | | 151 (98%) | 6 (4%) | <0.001# |
| **NRS-11-Pain** | **Day 1 – Rest** | | 2 (0-4) | 2 (0-4) | 0.281* |
|  | **Day 1 – Max.** | | 3 (1-5) | 3 (1-4) | 0.686* |
|  | **Day 2 – Rest** | | 2 (0-3) | 1 (0-2) | 0.045* |
|  | **Day 2 – Max.** | | 2.5 (0-5) | 2 (0-3) | 0.015* |
|  | **Day 3 – Rest** | | 1 (0-3) | 0 (0-2) | 0.049* |
|  | **Day 3 – Max.** | | 2 (0-4) | 0.5 (0-2) | 0.010* |
|  | **Discharge – Rest** | | 0 (0-1) | 0 (0-1) | 0.880* |
|  | **Discharge – Max.** | | 0 (0-1) | 0 (0-2) | 0.781* |
| **Peridural catheter** | | | 43 (28%) | 7 (5%) | <0.001# |
| **Days with PDK** | | | 0 (0-2) | 0 (0-0) | <0.001* |
| **Opiods at discharge** | | | 62 (41%) | 68 (44%) | 0.519# |
| **Time to First Bowel Movement (TTFBM) (days)** | | | 2 (2-3) | 2 (2-3) | 0.321* |
| **Length of Stay (days)** | | | 7 (5-9) | 5 (3-6) | <0.001* |
| **Length of ICU/IMC-stay (days)** | | | 0 (0-2) | 0 (0-0) | 0.020* |
| **ICU readmission** | | | 14 (9%) | 8 (5%) | 0.347# |
| **Clavien-Dindo-Classification** | | **Grade 0** | 106 (69%) | 128 (84%) | 0.011# |
|  |  | **Grade 1** | 16 (11%) | 8 (5%) |  |
|  |  | **Grade 2** | 13 (9%) | 5 (3%) |  |
|  |  | **Grade 3a** | 5 (3%) | 0 (0%) |  |
|  |  | **Grade 3b** | 7 (5%) | 11 (7%) |  |
|  |  | **Grade 4a** | 3 (2%) | 1 (1%) |  |
|  |  | **Grade 4b** | 2 (1%) | 0 (0%) |  |
|  |  | **Grade 5** | 1 (1%) | 0 (0%) |  |
| **Clavien-Dindo-Classification** | | **Grade 0-3a** | 140 (91%) | 141 (92%) | 0.835# |
|  |  | **≥ Grade 3b** | 13 (9%) | 12 (8%) |  |
| **Re-Operation** | | | 9 (6%) | 12 (8%) | 0.498# |
| **Seroma (req. Intervention)** | | | 5 (3%) | 1 (1%) | 0.099# |
| **Hematoma (req. Intervention)** | | | 3 (2%) | 6 (4%) | 0.310# |
| **Wound infection (req. Intervention)** | | | 6 (4%) | 1 (1%) | 0.056# |
| **Mesh infection (req. Intervention)** | | | 4 (3%) | 1 (1%) | 0.176# |
| **Blood transfusion** | | | 8 (5%) | 6 (4%) | 0.593# |
| **SSOPI** | | | 15 (10%) | 12 (8%) | 0.533# |
| **Re-Admission (30 days)** | | | 6 (4%) | 6 (4%) | 0.991# |

*Mann-Whitney-U-Test; #chi-squared-test/Fisher's exact test

**Table S4:** Patient satisfaction and postoperative Carolina Comfort Scale

|  | **Open-RS-cohort**  **(n=84)** | **eTEP-RS-cohort**  **(n=138)** | **p-value** |
| --- | --- | --- | --- |
| **Overall patient satisfaction with surgery and long-term-result** (1: Very dissatisfied, 2: Dissatisfied, 3: Neutral, 4: Satisfied, 5: Very satisfied) | 4.0±1.5 | 4.5±0.8 | 0.028* |
| **Overall Score Carolina Comfort Scale** (scale 0–115) | 4.4±9.8 | 3.6±12.2 | 0.820* |
| **CCS – Pain** (scale 0–40) | 2.5±7.0 | 1.7±5.6 | 0.017* |
| **CCS – Mesh Sensation** (scale 0–40) | 0.4±1.7 | 1.1±4.0 | 0.021* |
| **CCS – Movement** (scale 0–35) | 1.4±5.2 | 0.8±4.2 | 0.129* |

Carolina Comfort Scale (CCS): Mesh sensation, severity of pain, and movement limitation were collected as continuous measurements on a 6-point scale within the following functional actions: lying down, bending over, sitting, activities of daily living, coughing or deep breathing, walking, walking upstairs, and exercise. The CCS score is derived by adding the scores from each of the 23 items. The best possible score is 0 and the worst possible score is 115. mean±standard deviation; *Mann-Whitney-U-Test

Reference: Heniford BT, Walters AL, Lincourt AE, Novitsky YW, Hope WW, Kercher KW. Comparison of generic versus specific quality-of-life scales for mesh hernia repairs. J Am Coll Surg. 2008 Apr;206(4):638-44. doi: 10.1016/j.jamcollsurg.2007.11.025.
